# Multi-pollutant surface objective analyses and mapping of air quality health index over North America

**DOI:** 10.1007/s11869-015-0385-9

**Published:** 2016-01-07

**Authors:** Alain Robichaud, Richard Ménard, Yulia Zaïtseva, David Anselmo

**Affiliations:** 1 Air Quality Research Division, Environment and Climate Change Canada, 2121 Trans Canada Highway, Dorval, Québec H9P 1J3 Canada; 2 Canadian Meteorological Centre, Environment and Climate Change Canada, 2121 Trans Canada Highway, Dorval, Québec H9P 1J3 Canada

**Keywords:** Objective analyses, Air Quality Health Index, Pollutants, Ozone, Particulate matter, Nitrogen dioxide

## Abstract

Air quality, like weather, can affect everyone, but responses differ depending on the sensitivity and health condition of a given individual. To help protect exposed populations, many countries have put in place real-time air quality *nowcasting* and forecasting capabilities. We present in this paper an optimal combination of air quality measurements and model outputs and show that it leads to significant improvements in the spatial representativeness of air quality. The product is referred to as multi-pollutant surface objective analyses (MPSOAs). Moreover, based on MPSOA, a geographical mapping of the Canadian Air Quality Health Index (AQHI) is also presented which provides users (policy makers, public, air quality forecasters, and epidemiologists) with a more accurate picture of the health risk anytime and anywhere in Canada and the USA. Since pollutants can also behave as passive atmospheric tracers, they provide information about transport and dispersion and, hence, reveal synoptic and regional meteorological phenomena. MPSOA could also be used to build air pollution climatology, compute local and national trends in air quality, and detect systematic biases in numerical air quality (AQ) models. Finally, initializing AQ models at regular time intervals with MPSOA can produce more accurate air quality forecasts. It is for these reasons that the Canadian Meteorological Centre (CMC) in collaboration with the Air Quality Research Division (AQRD) of Environment Canada has recently implemented MPSOA in their daily operations.

**Electronic supplementary material:**

The online version of this article (doi:10.1007/s11869-015-0385-9) contains supplementary material, which is available to authorized users.

## Introduction

Air quality (AQ), like weather, can affect everyone, but responses differ depending on the sensitivity and health condition of a given individual. Breathing clean air is an important aspect of quality of life (European Environment Agency–World Health Organization (EEA-WHO) 2002). Policy makers require more and more detailed AQ information to take measures to improve or mitigate the impacts of AQ, while epidemiologists seek more accurate exposure estimates to evaluate the health risk (Van de Kassteele 2006). A large number of studies have been published describing the role of air pollution in inducing or exacerbating disease. Health effects related to air pollution include eye irritation, asthma, chronic obstructive pulmonary disease (COPD) heart attacks, lung cancer, diabetes, premature death and damage to the body’s immune, neurological, and reproductive systems (Pope et al. 2002; EEA-WHO 2002; WHO 2003; Sun et al. 2005; Ebtekar 2006; Pope and Dockery 2006; Georgopoulos and Lioy 2006; Institute for Risk Research 2007; Reeves 2011; Crouse et al. 2015). It has recently been estimated, using coupled climate-chemistry global models with concentration-response functions, that up to 3.7 million premature deaths occur annually worldwide due to outdoor air pollution as compared to a reference year before widespread industrialization, i.e. year 1850 (Silva et al. 2013). Similar results have been found by the Global Burden Study 2010 who places outdoor air pollution among the top 10 risks worldwide (Lim et al. 2012). According to a study by the Canadian Medical Association (CMA 2008), 21,000 premature deaths due to air pollution occurred in the year 2008 with 11,000 hospital admissions, 620,000 doctor visits at a cost to the Canadian society evaluated at more than 8 billion dollars. Therefore, it is important to provide effective tools for assessing the quality of the air at any given time and everywhere in Canada. However, it is impossible to get a comprehensive overview of pollutant concentrations over large territories based only on ground-based measurements (Van de Kassteele 2006). To achieve this task, data fusion of observations and models are required. Knowledge of multi-pollutant concentrations in near real time is the first step towards a total environmental risk monitoring system (Georgopoulos and Lioy 2006; Institute for Risk Research 2007). Since pollutants can also behave as passive atmospheric tracers, they can give information about their dispersion and provide links to synoptic and regional meteorological phenomena. Multi-pollutant surface objective analyses (MPSOAs) could also be used to build air pollution climatology, compute local and national trends in air pollutants, and detect AQ model systematic biases (see Robichaud and Ménard 2014a, thereafter RM14a). Finally, initializing numerical AQ models at regular time intervals with MPSOA can produce more accurate air quality forecasts (Blond et al. 2004; Wu et al. 2008; Tombette et al. 2009; Sandu and Chai 2011; Silibello et al. 2014; Robichaud and Ménard 2014b, thereafter RM14b). This is the motivation behind the implementation at Canadian Meteorological Centre (CMC) of the MPSOAs described here. Moreover, MPSOAs are considered an important tool for environmental surveillance since they provide users (e.g. public, air quality forecasters, and epidemiologists) with a more accurate picture of the true state of air quality in the form of geographical maps of chemical species. The pollutants under study here are ozone, particulate matter (PM), and nitrogen dioxide (NO_2_). Note that PM_2.5_ (particulate matter with aerodynamic diameter less than 2.5 μm) ozone and nitrogen dioxide (NO_2_) are also inputs to the Canadian Air Quality Health Index (AQHI) (Stieb et al. 2008).

### Air Quality Health Index

In order to inform risk management strategies for human exposure to air pollutants and smog, various air quality health indices have been developed in different parts of the world. The term smog has been utilized in scientific texts since the mid-twentieth century to denote the phenomena that ensues when polluted air is trapped near the Earth’s surface. However, nowadays smog refers to the photochemical mixture formed by hazardous pollutants especially ozone, PM_2.5_, nitrogen oxides (NO_x_), and volatile organic compounds (VOCs) (Jacobson 2002). Ground-level ozone and PM_2.5_ are the primary contributors to poor air quality in North America (EPA 2012). These pollutants are also the main constituents of smog and, together with NO_2_, form the basis of the Canadian AQHI which has been designed to take into account the combined impacts on health risk of exposure to a mixture of these pollutants (Stieb et al. 2008). The value of the AQHI and the corresponding health risk are provided in Table 1. The AQHI is a risk communication tool, especially targeted at vulnerable populations.

**Table 1:** Air Quality Health Index and its relation to health impact

Health risk	AQHI
Low	1–3
Moderate	4–6
High	7–10
Extreme	>10

*Source*: “Environment Canada-Air-AQHI categories and explanations.” Retrieved 20 June 2014

### Objective analyses of surface pollutants

Air quality measurements are in general accurate and unbiased at the measurement location, but interpolation may introduce important representativeness errors and lead to imprecise descriptions of air pollutant levels away from measurement sites. Model outputs, on the other hand, are less accurate and have biases but have complete coverage over the domain and integrate the physics, chemistry, and meteorology of air pollution. An optimal combination (known as optimal interpolation) of both information leads to a significant improvement of the coverage and accuracy of air pollution patterns and is referred to as objective analysis (OA) which is defined as a combination of information (i.e. information from observations and information from short-term forecast air quality models) which are carefully blended to minimize an objective criteria. Optimal interpolation (OI), as well as variational methods (3D Var and 4D Var), have been extensively utilized for objective analysis over the past decades (Kalnay 2003). Here, we extend the work presented in RM14a to other pollutants (NO_2_ and PM_10_) and propose a derived product which has added value, namely, an AQHI geographical mapping tool which provides near real-time description of the air quality health risk index everywhere and every hour over North America (except Mexico). Note that currently, the AQHI index is available only at few hundred observation points covering less than half of Canadian population. We will see, in this study, that the mapping using OA makes the AQHI available everywhere.

The mapping of AQHI requires three inputs which are the MPSOAs which provide users (e.g., public, air quality forecasters, and epidemiologists) with a more accurate picture of the true state of a given chemical species and also the interaction between pollutants through the AQHI formulation (see “Theory and methods” section). On the other hand, by monitoring analysis increments (correction to the model by observation; see second member of the right-hand side of Eq. 3), it allows the tracking of systematic model errors in a multi-variate way (correlation between increments of different chemical species, e.g., O_3_ and NO_2_ and PM_2.5_ and PM_10_). MPSOA can also help define the total environmental risk to a population (see Georgopoulos and Lioy (2006) and Institute for Risk Research (2007) for the concept of total environmental risk). Finally, MPSOA could be used to initialize air quality models which may improve model predictions (Blond et al. 2004; Tombette et al. 2009; Wu et al. 2008; RM14a,b). Optimal interpolation, as used here for objective analysis, is a robust and flexible method to perform data assimilation in air quality and has been shown to give comparable results to the more sophisticated methods such as 3D Var or even 4D Var for surface tracer such as ozone (Wu et al. 2008). In air quality, assimilation of hourly data is required since, unlike meteorology which is sensitive to initial conditions and where a noise filtering is used (e.g., due to the spin-up problem), pollutants are largely controlled by sources and sinks and boundary conditions as well as atmospheric conditions (Elbern et al. 2010).

One of the key components of data assimilation, or objective analysis, is error statistics. The latter directly influences the weight given to the different sources of information. The prescription of adequate error statistics for air quality can be challenging. Unlike the free troposphere or the stratosphere, boundary layer problems and complex topography and physical obstacles make it difficult to produce error covariance statistics for ground pollutants such as NO_x_ and particulate matter as well as ozone. The hypothesis that observation representativeness errors are isotropic and homogeneous is questionable at the surface. This is particularly true in mountainous regions (Tilmes 2001) or in urban environments (Bédard et al. 2015). On the other hand, atmospheric models show considerable uncertainty in the boundary layer and near surfaces (Reidmiller et al. 2009; Bosveld et al. 2014). This is especially true in complex orography where very high resolution models are needed to resolve small-scale features (Bernier et al. 2014). However, the relatively flat topography found over eastern and central North America and the importance of transport of ozone and PM_2.5_ and other medium to long-lived pollutants above and within the boundary layer make these pollutants excellent candidates for objective analysis and data assimilation. It is important that the correlation length be significantly larger than model resolution so that information can be spread around efficiently over more than one model grid point which is the basis of an intelligent interpolation.

## Theory and methods

In this study, Environment Canada’s air quality model, Global Environmental Multi-scale coupled with Model of Air quality and Chemistry (GEM-MACH) version 1.3.8.2, has been used to produce the “first-guess” forecast. The output of this forecast is blended with surface observations to produce the MPSOA. This air quality model is part of the Canadian Air Quality Regional Deterministic Prediction System (AQRDPS) with a spatial resolution of 10 km (Moran et al. 2012). The objective analysis exploits air quality surface observations from the US Aerometric Information Retrieval Now (AIRNow) program, as well as Canadian observations measured in real time by the provinces and territories (and some municipalities). Figure S1 (supplementary material S1a) depicts the flow chart of the production of the surface Regional Deterministic Air Quality Analysis (RDAQA) in an operational environment at CMC. The observations are acquired in real time (get_obs) and are combined with a first-guess model forecast (get_fcst). The observations are passed through a series of quality controls to check for (a) exceedances of maximum and minimum concentration values, (b) dubious hourly jump detection, and (c) background check of observed-minus-forecast increments (module background check and get_bgcksfc). Details of the quality control algorithm are given in supplementary material S1b. Optimum interpolation uses an exponential decay function over distance (see below), and a first estimate of the error statistics (error variance matrices) is obtained from the Hollingsworth and Lönnberg’s method (Hollingsworth and Lönnberg 1986, thereafter HL86; Lönnberg and Hollingsworth 1986). In RM14a, it was found that a scaling of both the correlation length (mostly deflation) and the background error variance (mostly inflation) had to be done in order to improve the performance of both the bias and error variance of the analyses whenever HL86 method was used. Furthermore, whenever HL86 method is inapplicable (whenever the data is too noisy or too many observations are missing), we have followed Silibello et al. (2014) (thereafter S14) with some modification to deduce background error variance (see below for more details). Although the error correlations are modeled as homogeneous and isotropic, the spatiotemporal variability of the background error variance is taken into account which reflects the intrinsic variability of the surface pollutant concentrations at a given station. A regional bias correction could also be applied for any pollutants depending on the situation (see below). The production of objective analysis is done in the module *analsfc* and is output as a four-panel product (submodule Four_Panel_Images in Fig. S1a). One question which arises at this point is how to interpolate spatial AQHI values to produce maps. The module AQHI computes the air quality index according to the following formula (Stieb et al. 2008):

1 $$ \mathrm{AQHI}=\frac{10}{10.4}*\left[\ 100*\right(\Big( \exp \left(0.000871*{\mathrm{NO}}_2\Big)-1\right) + \left( \exp \left(0.000537*{\mathrm{O}}_3\Big)-1\right) + \left( \exp \left(0.000487*{\mathrm{PM}}_{2.5}\ \Big)-1\right)\ \right)\right] $$

where NO_2_, O_3_, and PM_2.5_ are, respectively, the concentration vector obtained from MPSOA. AQHI is an environmental health indicator that uses a 3-h running average of pollutant concentrations to summarize health risk to the general public and to particular audiences (at risk populations). Since the quantities under the exponential brackets are very small, we may use the approximation exp(*x*) ~1 + *x* (valid for small values of *x*). Equation 1 could then be re-written as

2 $$ \mathrm{AQHI} \sim \left(0.871*{\mathrm{NO}}_2 + 0.537*{\mathrm{O}}_3+0.487*{\mathrm{PM}}_{2.5}\right)\ /10.4 $$

For AQHI values up to 10, the maximum error with this approximation is less than 2 % which is well below the error of the inputs and observation errors (see Table 2 for typical observation errors). Note that Eq. 2 means that AQHI is now a linear combination of three pollutants weighted by their risk factor. Note also that an extensive cross validation of AQHI will not be performed here as for classical pollutants since the cross validation of the three inputs (objective analyses for NO_2_, O_3_, and PM_2.5_) are already performed below and also because AQHI is almost a perfect linear combination of these three inputs for most values below of AQHI below 10 (as shown by Eq. 2).

**Table 2:** Description of method of measurements and typical issues with different instruments

	Ozone	PM_2.5_	PM_10_	NO_2_
Typical error	5 ppbv or higher^a^ Typical total error less than 15 %^b^	About 2 μg/m^3^ for TEOM-SES^a^ For other monitor, random error is 5–10 %	5–10 % random error Typical total error less than 15 %^b^	5–10 % random error^b^ Typical total error less than 15 %^b^
Typical measurement method	Ultraviolet absorption or chemiluminescence	Oscillating microbalance BAMS (Beta attenuation) or Thermo SHARP	Oscillating microbalance, gravimetric methods	Molybdenum converter
Typical instrument problem or issue	Ozone drift at times and zero-span contamination possible	Underestimation due to volatilization at heated inlet especially in winter (only for TEOM-SES)		NO_z_ interference (up to 30 % overevaluation for NO_2_) in rural areas in summer

^a^Fleming et al. 2003

^b^Luc White, Environment and Climate change Canada, personal discussion

### Mathematical formulation for optimal interpolation

Optimum interpolation is an objective analysis method that uses a linear combination of the background field and observations and optimized by minimizing the error variance using stationary error statistics. The solution of this optimization problem can be written in the following form (e.g., Bouttier and Courtier 2002; Kalnay 2003):

3 $$ {\boldsymbol{\mathsf{x}}}^a={\boldsymbol{\mathsf{x}}}^f+\mathbf{\mathsf{K}}\left({\boldsymbol{\mathsf{y}}}^o-\mathbf{\mathsf{H}}{\boldsymbol{\mathsf{x}}}^f\right) $$

${\boldsymbol{\mathsf{x}}}^f$ is the background field obtained from a short-term AQ forecast model, at a grid point *n*; $\mathbf{\mathsf{H}}$ is an operator that performs an interpolation from the observation space to the model grid point space (here we use a bilinear interpolation); ${\boldsymbol{\mathsf{y}}}_n^o$ is the vector that contain all the observations for a given station *n*; and $\mathbf{\mathsf{K}}$ is the Kalman gain matrix. $\mathbf{\mathsf{K}}$ contains the statistics which minimizes the analysis error (see Eq. 4 for the optimum expression for $\mathbf{\mathsf{K}}$). However, the computation in observation space indeed requires the inversion of a matrix. Note that in meteorology, error statistics in OI are stationary, whereas here for air quality, they are defined for every hour so the analysis (Eq. 3) is also available on hourly basis. This is thought to be an important improvement over classical OI and allows the analysis to capture the diurnal cycle of pollution. The main assumptions behind optimum interpolation in our context are reviewed in RM14a and will not be repeated here. The gain matrix expression $\mathbf{\mathsf{K}}$ which minimizes the analysis error and its components is given as (Kalnay 2003)

4 $$ \mathbf{\mathsf{K}} = {\displaystyle {\left(\mathbf{\mathsf{H}}\mathbf{\mathsf{B}}\right)}^T}{\displaystyle {\Big(\mathbf{\mathsf{H}}\left(\mathbf{\mathsf{H}}\mathbf{\mathsf{B}}\right)}^T} + \mathbf{R}\mathbf{\Big)}{}^{-1} $$

where we have adopted the following formulation (as in RM14a):

5 $$ \mathbf{\mathsf{H}}{\left(\mathbf{\mathsf{H}}\mathbf{\mathsf{B}}\right)}^T\ \left({\mathrm{k}}_1,{\mathrm{k}}_2\right) = {\upsigma}^{\mathrm{f}}\left({\mathrm{k}}_1\right){\upsigma}^{\mathrm{f}}\left({\mathrm{k}}_2\right) \exp \left\{-\left|\mathrm{x}\left({\mathrm{k}}_1\right)-\mathrm{x}\left({\mathrm{k}}_2\right)\right|/\mathrm{L}\mathrm{c}\right\} $$

6 $$ {\left(\mathbf{\mathsf{H}}\mathbf{\mathsf{B}}\right)}^T\ \left(\mathrm{i},\mathrm{j},{\mathrm{k}}_1\right) = {\upsigma}^{\mathrm{f}}\left(\mathrm{i},\mathrm{j}\right){\upsigma}^{\mathrm{f}}\left({\mathrm{k}}_1\right) \exp \left\{-\left|\mathrm{x}\left(\mathrm{i},\mathrm{j}\right)-\mathrm{x}\left({\mathrm{k}}_1\right)\right|/\mathrm{L}\mathrm{c}\right\} $$

are the components of the **K** matrix and the superscript *T* indicates the transpose matrix operator. Note that Eqs. 5 and 6 contain an exponential function which provides the mechanism for interpolation. **B** is the background error covariance matrix, and **R** is the observation error covariance matrix. It should be noted that each term is computed explicitly and does not require the storage of the background error covariance matrix. Furthermore, we assume that σ^f^(i,j) and L_c_ are constant throughout the domain (homogeneous assumption), whereas σ^f^(k_1_) and σ^f^(k_2_) are defined locally at each observation station k_1_ and k_2_. But only the covariance matrix (**H**(**HB**)^*T*^ + **R**) is stored and then inverted. All computations and the analysis itself are produced each hour in the RDAQA system. Note that the covariance matrix

7 $$ \mathbf{\mathsf{A}} = {\displaystyle {\mathbf{\mathsf{H}}\left(\mathbf{\mathsf{H}}\mathbf{\mathsf{B}}\right)}^T}+\mathbf{R} $$

is called the innovation matrix and needs to be inverted only one time per analysis. A Cholesky decomposition (Golub and Van Loan 1996, Sect. 4.2) is used for the computation. Finally, we shall note that **A** must be a symmetric and positive definite matrix (that is each individual eigenvalue must be positive).

### Observations

**Fig. 1:**
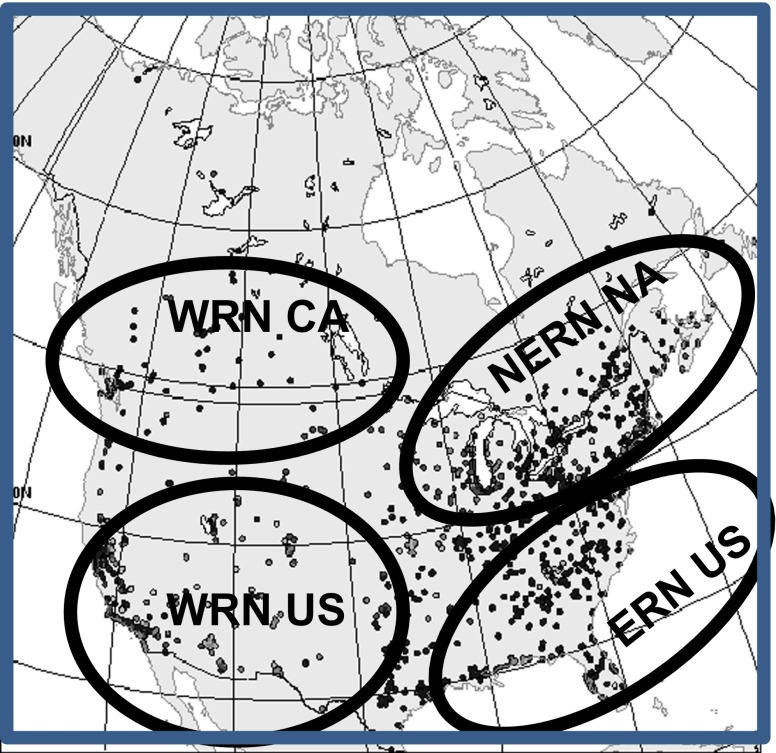
Location of reporting measurement sites. The number of sites available varies from one pollutant to another (see Fig. 3 for the reporting number of stations for each pollutant)

The observations utilized in the MPSOA are received at CMC and are rigorously quality-assured (see supplementary material S1b for details). How well observations represent the pollution concentration in a given region depends largely on local emission sources, topography and meteorology, boundary-layer characteristics, and the lifetime of the pollutant of interest. Therefore, the representativeness of a monitoring station should depend in some aspect on surrounding land use (see next section). Figure 1 shows the location of the monitoring sites used to produce MPSOA in the RDAQA system. The density of sites is high over the regions inside ellipses particularly in eastern USA and California (WRN USA) and the Gulf states and becomes lower elsewhere in the USA and southern Canada with little density in northern Canada. For the PM_2.5_, the number of sites is about two times less to that of ozone, although the geographical distribution of sites is fairly similar. PM_10_ observations are scattered in eastern USA, absent in eastern Canada, and dense only in the province of British Columbia (western Canada) and southwest USA and NO_2_, and observations are numerous only in southern Canada (except for Alberta which is well covered by monitoring stations) and scattered in USA. Typical measurements techniques for different pollutants are described in Table 2. Ozone is often measured by the mechanism of ultraviolet absorption according to the specification of US National Ambient Air Quality Standards (NAAQS)^1^. Observation error standard deviation (including representativeness errors) should not be less than 5 parts per billion by unit volume (ppbv) according to Fleming et al. (2003). In the case of PM_2.5_, the most common instrument is the Met-One Beta Attenuation Monitor (BAM) instrument and the Tapered Element Oscillating Microbalance (TEOM) which have been accepted as a standard^2^ since 1990 under NAAQS. On the other hand, large underestimations of measured concentrations due to volatilization have been noted in the past with the TEOM instrument (Allen et al. 1997; Allen 2010) mostly during the cold season (e.g., whenever the average daily temperature is less than 10 °C). Note, however, that since the beginning of 2013, no more TEOM-Sample Equilibration System (SES) is operating on a routine basis in Canada so the problem mentioned above is rarely an issue nowadays. Note, however, that in Montreal, TEOM-FDMS (FDMS stands for Filter Dynamic Measurement System) are still in use and systematically overreport by an error of 10 % (Luc White, NAPS, personal discussion, 2015). In the USA, the most commonly used instrument is the Met-One BAM or Thermo SHARP with TEOM-SES use becoming rare (Hanley 2014). Real-time US observations originate from a data repository centralized by Sonoma Tech (official mandatory for US EPA) in the context of the AIRNow program (www.airnow.gov). Raw data is provided by numerous US local air quality agencies (between 150 and 200 agencies in USA) as well as Canadian agencies (Table 3).^3^

**Table 3:** Number of air quality monitors in each region of Canada for each pollutant of the MPSOA

Region	O_3_	PM_2.5_	PM_10_	NO_2_
Atlantic	37	25	None	23
Quebec	50	47	None	22
Ontario	46	41	None	41
Prairies and North	48	47	8	47
Pacific and Yukon	31	51	24	34
CAPMoN	11	None	None	None
Total	223	211	32	167

### Bias correction

Bias correction is needed when the observations and model show systematic differences. The detection of bias is ideally made by comparing models and observations with independent data that are trusted as accurate and unbiased (Ménard 2010). It is, however, a complex issue, and many assumptions have to be made in order to simplify the problem since biases could originate from numerous causes (as pointed out by Dee and DaSilva (1998)). The treatment of bias correction adopted in this paper follows Robichaud and Ménard (2014a). Inside the regions defined by ellipses in Fig. 1, a spatially averaged bias correction applies, and outside these subregions, an exponential decrease of the average bias is imposed to avoid spatially abrupt changes of the bias correction. The reader is referred to Robichaud and Ménard (2014a) for more details.

### Air quality model (trial field)

The air quality model used in this study is GEM-MACH which is a limited area air quality operational model developed at Environment Canada. GEM-MACH is run online (chemistry online with meteorology), and its boundary is driven by the global meteorological model GEM (Côté et al. 1998a, b; Moran et al. 2012). The domain for the objective analysis is the same as the model domain and essentially covers North America with a spatial resolution of 10 km. It is important to understand that the trial field (or sometimes called the background or first guess) not only fills the gap between observations but also allows in the MPSOA context (through Eq. 3) an intelligent interpolation from the grid model space to the observation space since it permits the meteorology and chemical patterns to be preserved during the interpolation process. However, one of the weaknesses of the air quality model is that it is not initialized by chemical observations and hence could present large uncertainties associated with errors in emissions, boundary conditions, wind inaccuracies (speed and direction), atmospheric instability, solar radiation, characteristics of the boundary layer, precipitation, and uncertainties associated with chemistry parameterization (Reidmiller et al. 2009; Pagowski et al. 2010; Robichaud 2010; Moran et al. 2012; Zhang et al. 2012; Bosveld et al. 2014). If these models are constrained by chemical observations by using MPSOA, precision and reliability could be improved (Blond et al. 2004; Wu et al. 2008; Tombette et al. 2009; RM14b).

### Error statistics

**Fig. 2:**
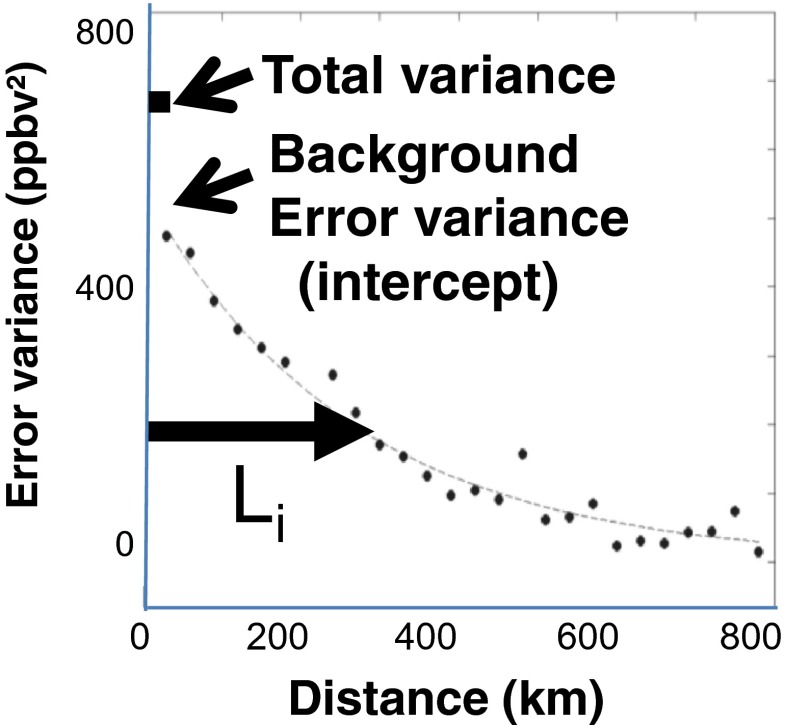
Evaluation method of error statistics parameter using H-L method. Background error *σ*_*B*_^2^ is given by the intercept of the curve computed using the FOAR model, whereas the correlation length L_c_ is given by the distance where this curve falls to 1/e of its value when the distance is zero (i.e., at intercept point)

**Fig. 3:**
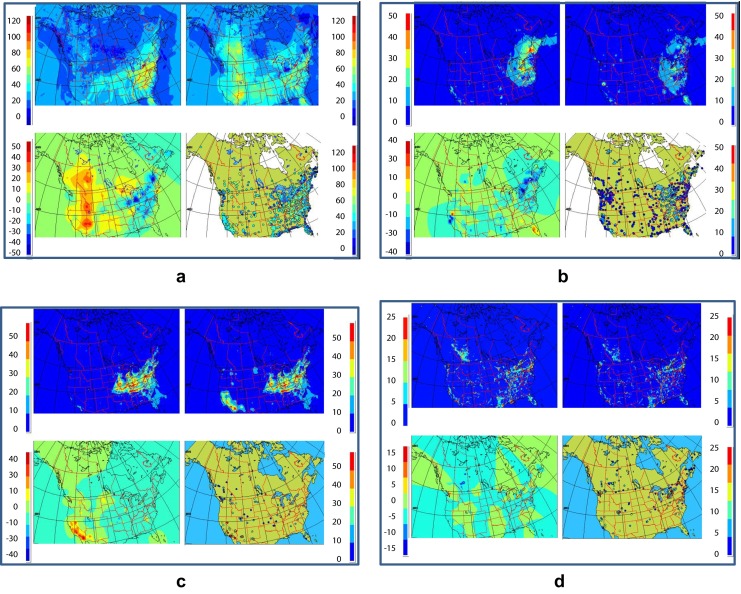
Example of the four-panel product for **a** ozone, **b** PM_2.5_, **c** PM_10_, and **d** NO_2_. In each product, we find the model forecast in the *top left*, the objective analysis in the *top right*, the analysis increments (correction to the model) in the *bottom left*, and the observations used to generate the analysis in the *bottom right*

Special attention should be given to the production of error statistics. Neglecting this may affect the optimality of an assimilation scheme (Daley 1991; Tilmes 2001; Bannister 2008a,b). The best source of information to compute error statistics are the innovations, i.e., the differences between model and observation (Daley 1991; Blond et al. 2004). The technique to compute error statistics from HL86 method follows these steps: (1) pairing up of different monitoring sites, (2) computing the covariance of Observation minus model Prediction (OmP) between the paired stations, (3) plotting the result as a function of distance with respect to the reference station, and finally, (4) fitting an homogeneous isotropic correlation model as a function of distance but excluding the data at the origin (see HL86; RM14a). Figure 2 shows an example of the application of the Hollingsworth and Lönnberg method for a typical site (here a site which lies in an area of high density of observing stations, that is the Goddard Space Flight Center air quality monitoring station): *σ*_*f*_^2^ is the intercept of the fitted first-order autoregressive model, and *σ*_*o*_^2^ is the residual (or nugget) error variance at zero distance. The fitting, called first-order autoregressive (FOAR, which is a simple exponential function), allows an estimation of the local isotropic correlation length L_ci_, at site *i*. Since the correlation model does not allow for non-homogeneous background error correlations, a spatially averaged uniform correlation length is used in the optimum interpolation computer code (i.e., σ^f^(i, j) is the average value through the whole domain in Eq. 6). Note that the elements of the background error covariance matrix **B** vary every hour to follow diurnal cycle and every season to capture seasonal changes. To summarize, the OmP is first obtained, and this quantity evaluated at observation location is a combination of model and observation error. The method HL86 allows to separate the former and the latter by using spatial autocorrelation of OmP. The part which is spatially correlated is associated to *σ*_*f*_^2^, whereas the uncorrelated part is associated with *σ*_*o*_^2^, the sum of the two parts giving the total variance of OmP obtained from observation and model data. The variogram shown in Fig. 2 is re-computed for all hours, all monitoring stations, and all seasons whenever possible. However, the HL86 method does not always produce successful FOAR models for background error variance statistics (e.g., intercept or correlation length, *L*, in Fig. 2) especially for the case of PM_10_, and NO_2_, since the density of stations is weaker as compared to ozone and PM_2.5_ (especially over USA; see right bottom panels of Fig. 3c, d). With a lower density of stations, fitting covariance models as shown in Fig. 2 to obtain error statistics becomes more challenging. Therefore, to overcome the difficulty of application of the HL86, the methodology proposed in S14 but modified as described below with the effective model resolution has been adopted,

8 $$ {\sigma}_0^2={\sigma}_{\mathrm{instr}}^2\left(1+\frac{n\varDelta x}{4{L}_{\mathrm{repr}}}\right) $$

where *σ*_*o*_^2^ is the observation error variance, *σ*_instr_^2^ is the instrument error variance, n*Δx* is the *effective* model resolution (where *n* is taken as equal to four times the model numerical resolution *Δx*, which is 10 km), and 4*L*_repr_ is the representativeness length associated with ground-based monitoring stations (values of 10, 4, and 2 km, respectively, for rural, suburban, and urban stations). Note that contrary to the procedure in S14, we did not use a tuning factor in Eq. 8. Instead, we choose the value *n* = 4 which is consistent with the concept of effective model resolution (i.e., 4*Δx*; see Pielke 2013). Moreover, using the value *n* = 4 is a consistent choice since it results in a representativeness error that is always greater or equal to the instrument error. In fact, we believe that for surface air quality, pollutant representativeness errors should normally be greater than instrument error. With *n* = 4, a mean ratio of about 3 between representativeness over instrument error variance was found using Eq. 8 which is consistent with Bédard et al. (2015), who found a similar ratio in the context of assimilation of surface winds (instrument error variance of 1 m^2^/s^2^ and representativeness of about 3 m^2^/s^2^). Note that the ratio of the effective model resolution over the representative length in Eq. 8 reflects the difficulty in capturing small-scale variability with interpolation techniques especially in urban environments where this variability could be large (i.e., urban gradients). The two methods (HL86 and S14) both have uncertainties. Therefore, blending results from the two methods for estimating *σ*_*B*_^2^ is likely to give more robust results, and this is the approach proposed here. The variance of the OmP (varOmP) computed with the residual of observation and model output is by definition the sum of the observation error variance and the background error variance *σ*_*B*_^2^, so we can deduce that

9 $$ {\sigma}_B^2 = \mathrm{varOmP} - {\sigma}_o^2 $$

where *σ*_*o*_^2^ is obtained from Eq. 8 when using S14 method. Note that in HL86 method, we compute *σ*_*o*_^2^ and deduce *σ*_*o*_^2^. Having two independent methods to compute error variances permits us to roughly estimate the uncertainty associated with *σ*_*B*_^2^. The mean values for *σ*_*B*_^2^ computed using Eqs. 8 and 9 versus HL86 differs by about 30–40 % in summer and 10–30 % in winter depending on the pollutant, with background error variance obtained from HL86 systematically lower than obtained through S14. Note that Eq. 8 with *n* = 4 (instead of *n* = 1 in S14) was found to give more consistent results when compared to the inflated error statistic procedure found in RM14a (i.e., 10–40 % inflation of background error variance required for best results). For the correlation length, fixed values are used but are allowed to vary depending on the pollutant, the hour, the season, and land use. Note that the correlation length found by using HL86 (see Fig. 2) was not used since it became obvious that the correlation obtained by the latter method was too long and a procedure of deflation was developed to correct it (see RM14a). A large uncertainty exists concerning the correlation length which is difficult to quantify from HL86 method. However, in the literature, it seems that the range for it would be from 10 to 100 km depending on the season, land use, hour, and pollutant type (Sandu and Chai 2011; S14; RM14a).

Combining model and observation gives better mapping. The objective analysis error variance *σ*_*a*_^2^ is always smaller than both the background error variance *σ*_*B*_^2^ and the observation error variance *σ*_*o*_^2^ (see Kalnay (2003) for a derivation), i.e.,

10 $$ 1/{\sigma_a}^2 = 1/{\sigma_B}^2+1/{\sigma_o}^2 $$

with *σ*_*a*_^2^ defined as follows:

11 $$ {\sigma}_a^2=<{\varepsilon}_a^n{\varepsilon}_a^n> $$

where *ε*_*a*_^*n*^ is the analysis error with the bracket <> indicating an average. The precision of the analysis (defined as the inverse of the error variance) is then the sum of the precision of the model (background) and the observations (Kalnay 2003). Therefore, according to Eq. 10, geographical mapping of pollutants is therefore more precise with an objective analysis (i.e., fusion of model and observation) than model and observations taken separately. Note that Eq. 10 was derived using the least squares method theory and there is no assumption whether the distribution needs to be Gaussian or not (Kalnay 2003). Simplifying for the scalar case (as opposed to the matrix formulation in Eq. 10) and re-arranging in terms of the gain of MPSOA over model gives

12 $$ {\sigma_a}^2\ /{\sigma_B}^2=1/\left(1+\kern0.5em \uplambda \right) $$

with λ = *σ*_*B*_^2^/*σ*_*o*_^2^, the ratio of background error variance over observation error variance.

### Validation

Objective analyses should have no biases, low random error, and high reliability. Three metrics are used to establish the performance of MPSOA and are defined in Appendix: (1) mean bias (average O − P or Observation minus Prediction and O − A Observation minus Analysis), (2) standard deviation of O − P and O − A to evaluate random error (i.e., root-mean-square error (RMSE)), and (3) frequency of being correct within a factor two (FC2) to assess reliability. We believe that these three metrics form a set of non-redundant metrics. Note that the metric FC2 is a more robust measure of the performance since it is not sensitive to “outliers” or “compensating errors” (Chang and Hanna 2004). Two types of validation are normally required, internal and external validation. The former verifies the coherence and detects gross error in the system, whereas the latter is considered as a true independent validation. Metrics utilized applied to all observations available for internal validation. In the case of external validation, a subset of observation (called independent observation) are withdrawn from the analysis computation and are used only to perform the independent validation (these observations are not seen by the objective analysis). This validation is also called cross validation and is similar to the “leaving one” concept. For ozone and PM_2.5_, we randomly select 90 % of the data to produce the objective analysis and 10 % of the data to perform the verification itself; the latter 10 % of observations was never used by the analysis. In the case of the remaining pollutants (PM_10_, NO_2_, and SO_2_), the ratio is taken as 75–25 %. A lower ratio is used for the latter pollutants (75 % as opposed to 90 %) because fewer observational data is available (especially in the USA; see Fig. 3c, d) so that a higher percentage of data is required for independent validation to obtain statistical significance with the tests. Three sets of experiments, selecting at random independent data, are normally enough to complete the external validation, and the results of the three sets are combined into a single test to achieve more statistical power. This combined test provides enough information so that a high degree of statistical confidence (i.e., *p* value < 0.05) exists for the results obtained. The verification is performed in the following four different regions similar to what was described before: eastern and western Canada and eastern and western USA with the longitude 90°W to delineate the boundary between east and west.

## Results

The new objective analyses are presented as a four-panel image. Figure 3a–d shows the results of the proposed (RDAQA system) for ozone, PM_2.5_, PM_10_, and NO_2_, respectively. For each pollutant, the four-panel image provides the model trial field in the top left panel, OA in the top right (fusion of model and observations), and analysis increments in the bottom left (or correction to the model computed by the second term on the right-hand side of Eq. 3). Finally, in the bottom right panel, observations used in the analysis are presented. The units are in ppbv for ozone and NO_2_ and in units of μg/m^3^ (micrograms per cubic meter) for PM_2.5_ and PM_10_. It is important to note that at the resolved scale (approximately four times the numerical resolution of 10 km, i.e., effective resolution of about 40 km for OA and model grid), certain local conditions such as titration of ozone by NO_2_ (due to local traffic), individual point sources such as pollution plumes originating from chimneys (in the case of PM_2.5_), or from other point source are neither correctly resolved by the model nor the analysis.

### Monitoring of MPSOA (internal validation)

Monitoring of OA (internal validation) is described in the supplementary material S2, whereas cross validation (external, i.e., independent validation) is presented below. Figure S2a–d shows results of the monthly verification scores for ozone, PM_2.5_, PM_10_, and NO_2_, respectively. The verification scores of mean bias for O − P (lower curve labelled Mean OmP) and O − A (lower curve Mean OmA) and standard deviation for O − P (upper curve Std OmP) and O − A (upper curve Std OmA) appear as a function of hour (UTC). As well, the number of stations ingested is plotted in the bottom panel (maximum number of possible stations is currently near 1200 for ozone). In all cases, one can easily detect a reduction of the standard deviation scores for OmA (OA analysis) by an approximate factor of up to 2 (therefore, a reduction of the error variance by a factor of 4) and a strong reduction to near zero for the bias in the analysis (OmA bottom curve) compared to that of the first guess (model forecast OmP). Note that the biases for all pollutants are reduced to zero for the analysis. Table 4 shows the performance for the FC2 metric, for (a) the summer period and (b) for winter. Results suggest that the model and analysis are very reliable for ozone during the period of 15:00 through 00:00 UTC (afternoon and evening) but less reliable from 03:00 to 12:00 *Z* (nighttime and morning) in all seasons. For all hours of the day, the analysis is always more reliable than the model (FC2 scores are closer to 1). For other pollutants, although FC2 are lower than that for ozone, the score for the analysis is always higher than that for the model. In other words, analyses are always (winter or summer and at any hour of the day) an added value over already existing information (observation or model).

**Table 4:** FC2 validation for selected hours (in UTC) for analysis and model for (a) July and (b) January

A		O_3_	PM_2.5_	PM_10_	NO_2_
00:00 *Z*	M	0.94	0.45	0.28	0.42
	A	0.99	0.78	0.92	0.70
03:00 *Z*	M	0.80	0.48	0.38	0.49
	A	0.93	0.78	0.88	0.68
06:00 *Z*	M	0.68	0.50	0.45	0.48
	A	0.85	0.79	0.88	0.66
09:00 *Z*	M	0.60	0.50	0.45	0.48
	A	0.80	0.77	0.85	0.62
12:00 *Z*	M	0.60	0.52	0.49	0.52
	A	0.80	0.78	0.90	0.70
15:00 *Z*	M	0.85	0.50	0.48	0.50
	A	0.96	0.81	0.91	0.75
18:00 *Z*	M	0.95	0.47	0.28	0.40
	A	0.99	0.80	0.93	0.70
21:00 *Z*	M	0.96	0.54	0.30	0.29
	A	1.00	0.78	0.92	0.69
B		O_3_	PM_2.5_	PM_10_	NO_2_
00:00 *Z*	M	0.80	0.48	0.46	0.60
	A	0.92	0.75	0.82	0.83
03:00 *Z*	M	0.68	0.48	0.50	0.61
	A	0.82	0.70	0.80	0.81
06:00 *Z*	M	0.66	0.45	0.50	0.60
	A	0.80	0.72	0.83	0.81
09:00 *Z*	M	0.66	0.48	0.48	0.58
	A	0.80	0.72	0.83	0.81
12:00 *Z*	M	0.68	0.45	0.50	0.60
	A	0.80	0.71	0.81	0.83
15:00 *Z*	M	0.75	0.45	0.50	0.60
	A	0.82	0.75	0.81	0.85
18:00 *Z*	M	0.89	0.40	0.49	0.58
	A	0.93	0.80	0.85	0.85
21:00 *Z*	M	0.94	0.40	0.45	0.60
	A	0.98	0.81	0.85	0.85

*M* model, *A* analysis

### Cross-validation tests (external validation)

**Fig. 4:**
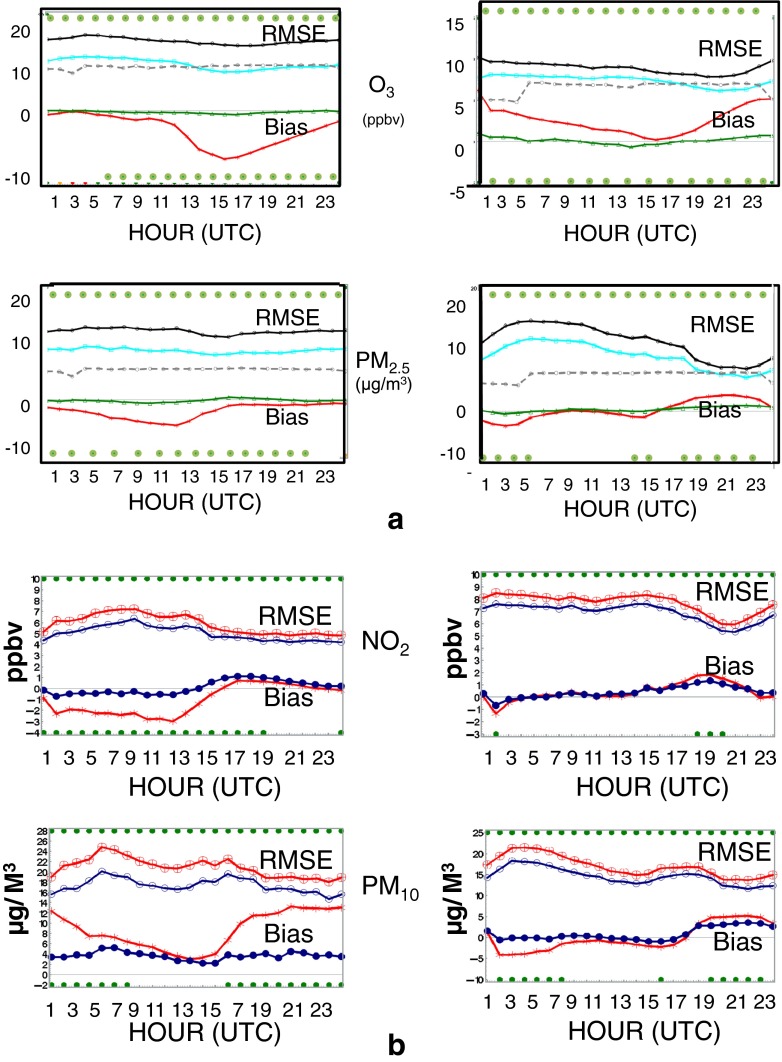
**a** Cross validation for ozone and PM_2.5_ and **b** for PM_10_ and NO_2_. Whenever a *green dot* appears in the *upper part* of each of the graphs, it indicates significant difference with a level of confidence greater than 95 % for the RMSE (standard deviation O − A vs O − P). Similarly, *green dots in the lower panels* indicate significant differences for biases

As mentioned above, cross validation for pollutants consists of reprocessing the objective analysis but with a subset of the data to produce OA outputs leaving out the remaining data to perform the verification itself. Figure 4a shows the results for ozone and PM_2.5_ for the period of July 2011 (left) and January 2012 (right). As in the internal validation, mean OmP and OmA measure biases, and standard deviation of OmP/OmA measures root-mean-square error (see Annex A for more details). One set of curves (red and black) show the performance of the model only and the other (green and cyan) the performance of the OA. This shows the added value of objective analyses (O − A curves) over model error (O − P curves). A very significant reduction of both errors (systematic and random) is obtained with the objective analyses at almost any time of the day as compared to the model forecast (as in the internal validation mode). Note that whenever a green dot appears on top (Fisher’s test for the variance) and/or bottom (*t* test for average) for a specific hour, it means that the model error (OmP) versus MPSOA error (i.e., OmA) is significantly different at the level of confidence exceeding 95 % (*p* value < 0.05). Success of the cross validation suggests not only that the methodology presented in the previous sections is sound but also implies that OA yields reasonably precise values in areas where there are no observations (but not too far away from other surrounding monitoring sites). This builds a case for using OA in several applications where geographical mapping is required but observations are often missing or do not fill the whole domain (e.g., useful for a climatology map of pollutants with OA or calculating trends from OA rather than directly using only observations; see RM14a). For other pollutants (PM_10_ and NO_2_; Fig. 4b), and for other years (July 2014 and January 2015), similar results were obtained and similar conclusions can be drawn as for ozone and PM_2.5_.

### Application of MPSOA

MPSOAs are important to provide users (policy makers, public, epidemiologists, and air quality forecasters) with better tools for understanding spatial and temporal trends in air quality. Another potential application of this work is the initialization of numerical air quality models at regular time intervals with MPSOA which may improve the predictive capacity of these models. Some experiments have been done which use the MPSOA produced in this study to initialize AQ models. To date, these experiments have shown encouraging results for improving short-term AQ forecasts (RM14b). MPSOA could also be used to produce air pollution climatology, compute long-term trends (RM14a), and provide long series of surface pollutants analyses that could be used by epidemiologists.

### Mapping AQHI over Canada and USA

**Fig. 5:**
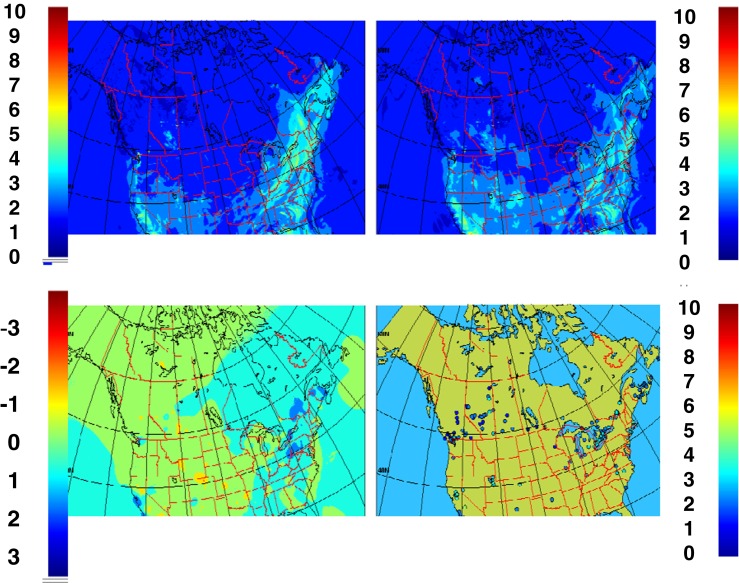
Same as Fig. 3 but for AQHI. Note that for the pseudo analysis AQHI, residuals are provided instead of analysis increment (as for individual pollutants) and the inputs to the AQHI analysis are MPSOA (not local AQHI observations). Note also that AQHI is unitless

In this section, we provide a first prototype of geographical mapping of AQHI in Canada and the USA based on data fusion and MPSOA (Fig. 5). It uses as input to Eq. 1, the objective analyses of ozone, PM_2.5_, and NO_2_ in the matrix form to produce a geographical map for AQHI. Since AQHI mapping is derived from the OAs for ozone, PM_2.5_, and NO_2_, we consider this mapping to be a *pseudo objective analysis* for AQHI (because it is derived from objective analyses for ozone, PM_2.5_, and NO_2_ using Eq. 1) and not from Eq. 3 as in the classical sense. Note that prior to operational implementation of our work at CMC, Eq. 1 was applied locally only at the observation point (at the sites shown at right bottom panel of Fig 5) and AQHI was available to less than 80 % of Canadians (mostly at specific urban centers; see the right bottom of Fig. 5 for locations where AQHI was currently available to the Canadian public). In the USA, at the current moment, official air quality index mapping is available everywhere but the methodology is based on krigging of observations (interpolation using inverse distance weighting; see Shepard 1968). The US mapping does not involve data fusion and uses only observations. With the AQHI mapping as proposed here, which is based on data fusion of models and observations, the interpolation takes into account physics, chemistry, and meteorology (as provided by the model). Moreover, an AQHI value becomes available anywhere in Canada and even for most of the USA (upper right panel in Fig. 5) and, in principle, the maps always provide full spatial coverage (contrary to AQHI site-specific values previously publically available in Canada but based on observations alone; see right bottom panel of Fig. 5). This mapping as presented here is therefore considered as a substantial improvement for environmental monitoring and management of health risk in Canada and USA and could be easily extended to any areas of the world.

**Fig. 6:**
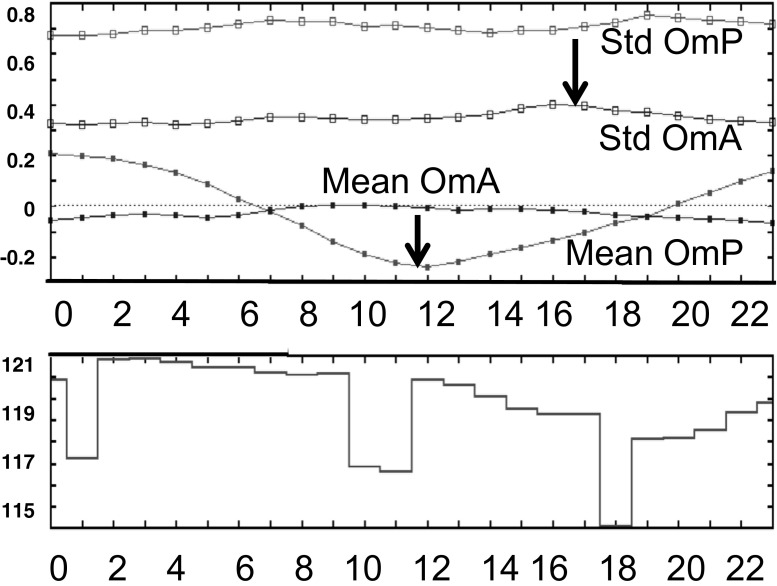
Validation of AQHI using Eqs. 13 and 14 (See Annex 1). The *black arrows* indicate the reduction of the RMSE (i.e., std dev.) error (*top arrow*) and the bias error reduction (*bottom arrow*) found from model (OmP) to analysis (OmA)

The validation of this OA-AQHI was done as follows. It consisted of using an observation at a particular site and comparing it with AQHI values obtained from Eq. 1 using real observations for verification instead of MPSOA matrices as input. We also used the AQHI model calculation as a reference and also compared the model calculation of AQHI with observations of AQHI (OmP). Hence, these two sets of values for OmP (model) and OmA (OA_AQHI) were available and compared each other. Figure 6 shows the results of such validation of the *pseudo* analysis of AQHI compared to AQHI computed from the model. The OmA biases and RMSE (mean and root mean squared error for AQHI) are greatly reduced as compared to that of OmP (model computation of AQHI). Differences between OA-AQHI and observations (OmA curve) and AQHI calculated from the model and observations (OmP curve) are greatly reduced for both biases (lower curves) and random errors (upper curves). The black arrows on the figure indicate the RMSE and bias reductions obtained by MPSOA.

### Statistics and climatology of AQHI

**Fig. 7:**
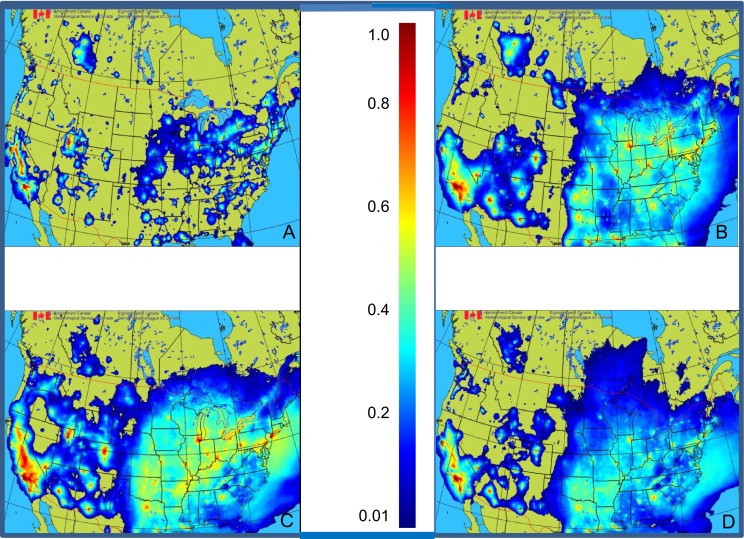
AQHI climatology for 2013: percentage of hours that AQHI index was superior to 3 (lower threshold for moderate to elevated risk to health due to air pollution) for **a** winter (DJF), **b** spring (MAM), **c** summer (JJA), and **d** autumn (SON). Note that whenever the background geography is seen (with no color from the color bar), it means that the frequency of the number of hours that AQHI is less than 3 is inferior to 1 % (i.e., unpolluted zones)

Mapping AQHI statistics could be view as an integrated index which reveals where most frequently the classical pollutants (ozone, PM_2.5_, and NO_2_) are present to a level where they can pose a risk to health. Figure 7 shows for different seasons of the year 2013 the number of hours of exceedance of AQHI over the value of 3 (considered as a threshold for health risk; see Table 1). Hot spots of elevated AQHI are found in Central Valley (California) and eastern parts of USA in any seasons. In Canada, the corridor Windsor-Québec City and Alberta (Edmonton-Calgary Corridor and the oil sands area) are considered the hot spots. Note that overall, AQHI values are highest in spring and summer and AQHI values are sensitive and also impacted by meteorological short-term and inter-annual fluctuations. Note that the lack of photochemistry in winter and the corresponding reduction of type II pollution explain lower values of AQHI during that season (DJF in Fig. 7).

## Discussion

### Relation between atmospheric lifetime, error statistics, and MPSOA performance

By inspection of Fig. 4a, b, we observe a greater reduction of RMSE (i.e., std. dev. of OmP vs OmA) as well as biases (mean of OmP vs OmA) for surface pollutants having larger lifetime (such as ozone and PM_2.5_) as compared to species having shorter lifetime (e.g., NO_2_). Moreover, Table 4 which compares the FC2 metrics for both model and analysis also shows much higher values for that metric for pollutant with larger lifetime. To explain this, let us inspect on one hand the ratio of first-guess error variance (i.e., background error variance) over observation error variance (i.e., λ = *σ*_*B*_^2^/*σ*_*o*_^2^) and, on the other hand, the correlation length (L_c_). Both largely control the behavior of the objective analysis (see Eqs. 5, 6, and 12). According to Eq. 12, a larger value of λ implies a larger gain of the analysis error over model or background error variance (*σ*_*a*_^2^/*σ*_*B*_^2^) and vice versa. Data for error statistics obtained from the HL86 method as well as from Silibello et al. (2014) and used in our study show that for ozone and particulate matter, λ is typically about 2, whereas for NO_2_, this ratio is typically about unity. This means that for the long-lived pollutants, the analysis error variance will be expected to be reduced roughly by a factor of 3. For short-lived species, the ratio would be about two according to Eq. 12. Therefore, reduction of RMSE (or standard deviation) will be expected to be reduced by the square root of 3 (~1.7) for large lifetime pollutant and by square root of 2 (~1.4) for shorter-lived species. This is consistent with RMSE reductions observed in Fig. 4. On the other hand, correlation length is longer for long-lived species as compared to the short-lived species, and this influences how biases are reduced. Our experience indicates that biases are more easily corrected in the case of long-lived as opposed to short-lived species as can also be seen in Fig 4. Finally, the gain of objective analysis with respect to the model (*σ*_*a*_^2^/*σ*_*B*_^2^) is slightly higher in summer than winter for all pollutants likely due to shorter correlation length in all cases and due to less atmospheric mixing causing model representativeness error to be higher in winter (see Fig. 4 summer vs winter case).

### Uncertainties associated with OA

The typical correlation length utilized for the mapping in this study (~20–100 km for surface pollutants) is largely inferior to those obtained by the use of HL86 method (see Fig. 2). RM14a have suggested a deflation procedure to reduce the correlation length obtained from HL86 method. In our work, values used for the correlation length are in agreement with those suggested in the recent literature for surface assimilation of chemicals in Europe (see S14) or with those utilized in the US assimilation system with the CMAQ model (about 60 km; Sandu and Chai 2011). Nevertheless, there is still a great deal of uncertainty related to the optimum correlation length which needs to be resolved, and future work will address this. A general theory is needed to improve the determination of correlation length near the surface for chemicals. Comparing to S14, it is very likely that the HL86 method provides correlation lengths which are too long and background error variances *σ*_*B*_^2^ which are likely too small. The latter statement is supported by RM14a who found that a deflation procedure for correlation length combined with an inflation procedure for *σ*_*B*_^2^ would give optimum results when compared to independent observations. Concerning the ratio of observation error over background (model) error, we have found a typical ratio of about 1/3 for ozone as an example. According to Van de Kassteele (2006), for gas, observations have an error of 5–10 % whereas the model predictions about 20 % which gives a ratio of 1/3. This suggests that our results are in the right ballpark.

To reduce uncertainty, assumptions of homogeneous and isotropic for the optimal interpolation should be revised. For example, tight urban-scale pollution gradients or irregular topography and a low density of monitoring stations are three factors that create conditions that can result in difficulties in the validity of the above assumptions. However, early tests which used an inhomogeneous OA did not improve the results and were therefore abandoned. Nevertheless, for ozone and PM_2.5_, the fact that the density of the data over the USA and southern Canada is high (see Fig. 1), at least in urban centers and for some cases near coastline stations (California and US eastern seaboard), make the assumption of homogeneity and isotropy not critical at least for these locations. In the future, we will intent to derive inhomogeneous and anisotropic OA. The reader is referred to Frydendall et al. (2009) which proposed a treatment of anisotropy for chemical surface assimilation and to Blond et al. (2004) for non-homogeneous treatment of error statistics for air quality assimilation.

The primary goal of MPSOA and the pseudo analysis of AQHI is to provide a tool for understanding the spatial distribution of pollutants to assess air quality across Canada as mentioned above. The new product supports human health risk assessment and communication and cumulative environmental impact assessments. On the other hand, daily inspection of MPSOA reveals that imprints of meteorological patterns (wind patterns, temperature, fronts, precipitation area, thermal inversion, etc.) are often visible in the structure of the air quality model outputs and resulting analyses (e.g., Fig. 3). This is due to the fact that meteorology controls or drives chemical tracers and their transport (Jacobson 2002; Stohl et al. 2002; Stohl et al. 2003; Pagowski et al. 2010) and promotes photochemical transformations (Seinfeld and Pandis 1998). On a daily basis, we have observed very significant analysis increments especially for PM_10_. The explanation could be linked to model deficiency such as lack of windblown dust which is reflected in high analysis increments in southwest USA. Hence, MPSOA could also reveal model’s weakness.

## Summary and conclusion

Air quality, like weather, affects everyone but quite differently depending on the sensitivity and health condition of a given individual. High-resolution MPSOAs are important since they provide users (e.g., policy makers, public, epidemiologists, and air quality forecasters) with a more accurate and detailed picture of the true state of a given chemical species as compared to mapping based on observations or model output alone. Knowledge of multi-pollutant concentrations in near real time is a step towards a total environmental risk monitoring system. Models are generally characterized by known deficiencies for prediction of many pollutants, whereas measurement systems suffer from representativeness problems and lack of sufficient coverage and, thus, are often best suited to providing local air quality information. However, the OI technique, used in operational meteorology for decades, provides an optimal framework to extract the maximum information of both model predictions and observations (Rutherford 1972; Daley 1991; Kalnay 2003; Brasnett 2008). The OA used in this study combines model outputs from the Canadian air quality forecast suite with the US and Canadian observations from various air quality surface monitoring networks. The analyses are based on an OI with an explicit bias correction for pollutants (ozone, PM_2.5_, PM_10_, and NO_2_). The estimation of error statistics has been computed using a modified version of the HL86 and the use of the work of Silibello et al. (2014) to compute observation error variance whenever HL86 was not applicable (too noisy or sparse density of stations). Based on the results obtained in RM14a, using a *χ* ^2^ (chi-squared) diagnostic (Ménard and Chang 2000), the correlation length obtained by HL86 method was found to be too long and needs to be deflated. Better results were found using a prescribed correlation length similar to that prescribed by S14 which is also consistent with Sandu and Chai (2011) who uses a fixed value of 60 km for correlation length for ozone. Successful cross-validation experiments were performed with an OA setup using a subset of observations to build the objective analyses and with the remainder left out as an independent set of data for verification purposes. A new operational product (called RDAQA) has been implemented at CMC. These analyses fill a gap in the operational suite at CMC.

### The RDAQA system uses MPSOA which is a useful product for the following reasons:

1. MPSOA provides a more scientifically robust technique for mapping surface air quality data in North America. Cross-validation tests (Fig. 4) demonstrate that the error variance is reduced in a very significant way (up to a factor of 2 for the variance) and that the bias, with respect to observations (O − A), is reduced to near zero in the analyses as compared to the model forecast bias (O − P). Objective analysis is, thus, accurate (low O − A residual variances), reliable (expressed by a high value of FC2 index), and unbiased source of air quality information in places where there is no observational data (provided a sufficient density of measurement stations exists in the neighborhood).
2. As a risk management tool, the AQHI mapping using Eq. 1 (based on the formula from Stieb et al. 2008) provides better and more accurate real-time exposure information overall and therefore helps moving towards the concept of evaluation of environmental risk anywhere and anytime for Canada.
3. AQHI statistics as form of climatology make it easier for policy makers and epidemiologists to relate exposure to pollution to short- and long-term health risk.
4. From a scientific point of view, these objective analyses provide an improved tool for *nowcasting* of air pollution and, potentially, a better means of initializing numerical AQ models (through data assimilation) which could translate into better air quality forecasts.

### Electronic supplementary material

Below is the link to the electronic supplementary material.

ESM 1 (DOCX 443 kb)

ESM 2 (PPTX 260 kb)

## Appendix. Definition of metrics

Given **O** = {*O*_*i*_} and **X** = {*A*_*i*_} or {*P*_*i*_}, respectively, the observed pollutant concentrations, the objective analysis, and the interpolated forecast *F* at the point of observation of an ensemble of measurements stations *N*, *i* = 1,2,…,*N*, the following metrics are defined:

Bias (*B*) :

13 $$ B=\frac{1}{N}{\displaystyle \sum_{i=1}^N\left({O}_i-{X}_i\right)} $$

In the text, the bias is presented as Mean (OmP) or Mean (OmA) for model prediction and analysis, respectively.

RMSE and standard deviation of O − X:

14 $$ \mathrm{S}\mathrm{T}\mathrm{D}.\kern0.5em \mathrm{D}\mathrm{E}\mathrm{V}.=\sqrt{\frac{1}{N-1}{\displaystyle \sum_{i=1}^N{\left\{\left({O}_i-{X}_i\right)-\left(\overline{Oi- Xi}\right)\right\}}^2}} $$

In the text, the RMSE is presented as std. dev. (OmP) or Std (OmA) since for large *N*, both RMSE and standard deviation are equivalent.

FC2 (frequency of value within a factor two compared to observations):

15 $$ \mathrm{F}\mathrm{C}2=\frac{H}{N}\times 100 $$

where *H* is the count when the ratio *O*_*i*_/*X*_*i*_ is within the range of 0.5–2 and *N* is the number of total observations used in the analysis. Note that Eqs. 13, 14, and 15, respectively, evaluate the systematic bias, the random error, and the reliability. Validation is performed at each hour (00:00 to 23:00 *Z*) unless stated otherwise.

## Footnotes

1
www.epa.gov/air/ozonepollution/actions.html

2
www.epa.gov/particles/actions.html

3 In Canada, air quality monitoring is under a provincial jurisdiction and managed by Environment Canada as a form of partnership. For example, a network such as National Air Pollution Surveillance (NAPS) is the result of a federal-provincial and even municipal partnership (such as in the case of Montreal (MUC) and Metro Vancouver). A summary of the spatial distribution of stations in Canada is provided in Table 3
