# Supplementary material for: Multi-pollutant surface objective analyses and mapping of air quality health index over North America
Source: Air Qual Atmos Health. 2016 Jan 7;9(7):743–59. doi: 10.1007/s11869-015-0385-9 (PMC5054062; doi:10.1007/s11869-015-0385-9)
Supplement: Supplementary file 1 — (DOCX 443 kb) [file 11869_2015_385_MOESM1_ESM.docx]

**S1A. Supplementary information. Flowchart of the RDAQA system to produce MPSOA**

**
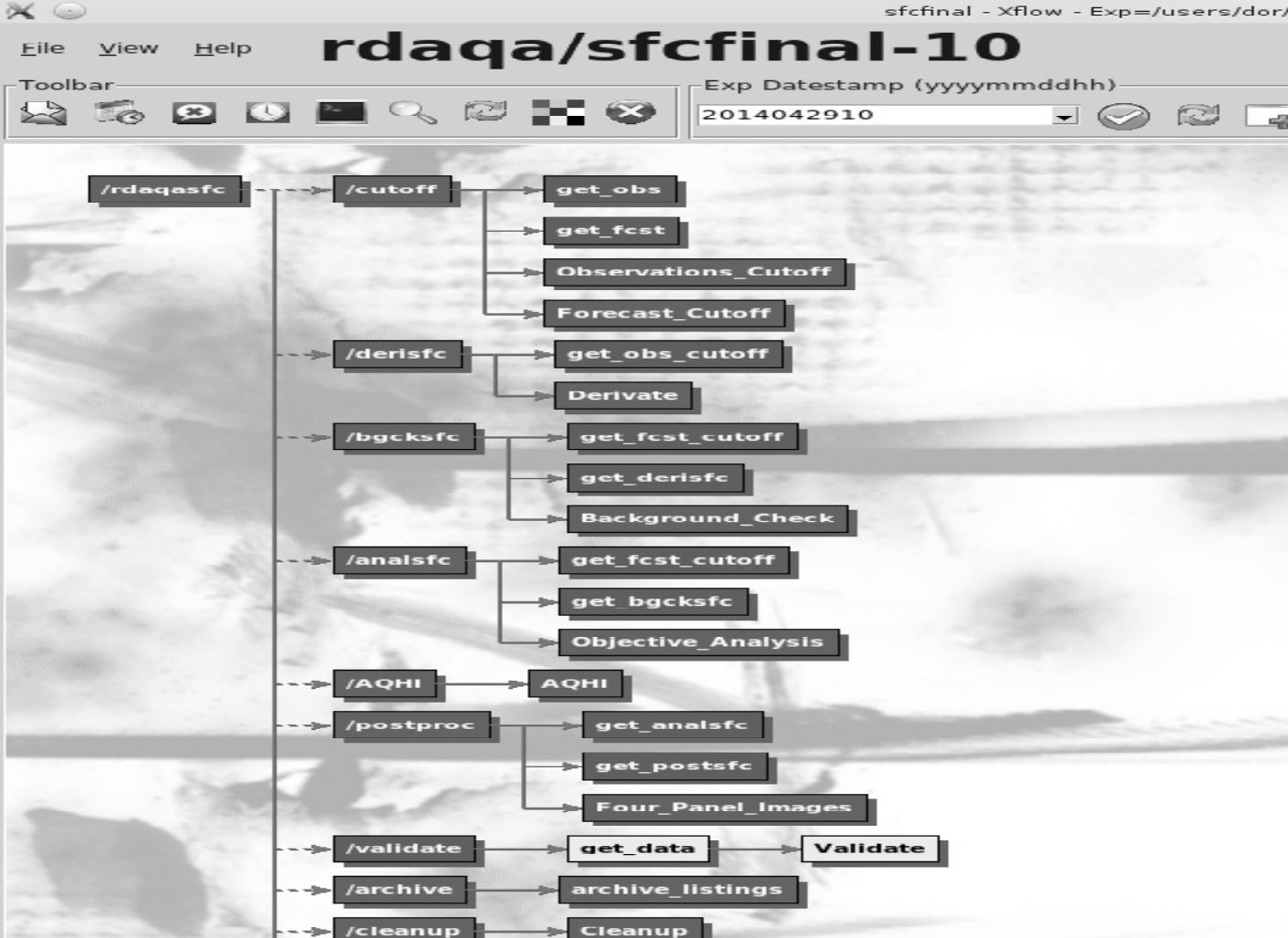
**

**S1B. Supplementary information. Details of quality control (QC) tests**

**First QC – QC done in the decoder**

1. **QC done for observations CAN**
2. **Test for observations already pre-validated**

Each Canadian observation is received from provincial or territorial agencies with a QC value already assigned, that is G,B,E,K,R,M (G (good), B(bad), E(estimated), K,R(suspect), M(missing)). After receiving at CMC, these QC values are used to change the observation FLAGS:

FLAG = FLAG|0 if GOOD (G), ESTIMATED(E) => (no bit switched on)

FLAG = FLAG|8 if SUSPECT (K,R) => (bit3 = 2**3 = 8)

FLAG = FLAG|2 if BAD (B) => (bit2 = 2**1 = 2)

1. **QC done for US observations**

In the case of US observations, those are already pre-validated in the context of AIRNow program. Therefore, no QC is done at this level.

**Second QC – QC done with a specialized program for observations**

1. **Threshold test (suspicious threshold, rejected threshold)**

This test is applied on both Canadian and US observations and on all pollutants.

Algorithm description:

If (OBS_CURRENT < min_error or OBS_CURRENT > max_error)

- then OBS_CURRENT is BAD

Elseif (OBS_CURRENT < min_suspect or OBS_CURRENT > max_suspect)

- then OBS_CURRENT is SUSPECT

Endif

MIN/MAX values (suspect), MIN/MAX (reject) criterias are pre-defined as follow :

**pollutant** **min threshold error min_suspect max_suspect max_error**

O_3_ -5.0 ppbv -5.0 200.0 500.0

NO_2_ -5.0 ppbv -5.0 100.0 2000.0

PM_2.5_ -10.0 ug/m^3^  -10.0 300.0 1000.0

PM_10_ -10.0 ug/m^3^  -10.0 300.0 1000.0

SO_2_  -5.0 ppbv -5.0 100.0 2500.0

NO -5.0 ppbv -5.0 100.0 2500.0

In very clean atmosphere, negative number could be reported at times. These data may be in fact valid and should not be flag as bad data or deleted. Moreover, correcting to zero would lead to biasing data higher (Hanley, 2014).

These QC values are utilized to change the FLAG value of observation:

FLAG = FLAG|8 if SUSPECT => (bit3 = 2**3 = 8)

FLAG = FLAG|2 if BAD => (bit2 = 2**1 = 2)

1. **Sudden jump test**

The sudden jump test is applied on both Canadian and US observations and is done only if the label GOOD appears. Only Ozone, PM_2.5_ and nitrogen dioxide are covered for the moment. The test is conceived to catch-up only an isolated single peak. For example, in the case of ozone, a peak sometimes comes from the auto-calibration « zero-span » which has not been filtered. If a peak spans over a longer period than one hour, it will not be flagged here but it is likely to be in the background check which follows later.

Algorithm description:

If (OBS_CURRENT is not GOOD)

- No action

If (OBS_CURRENT is GOOD) and (OBS_PRECCEDING is GOOD)

- If Then | OBS_ACT - OBS_PREC | > range**_jump** , then OBS_CURRENT is SUSPECT

If (OBS_CURRENT is GOOD) and (OBS_PRECEDENTE is not GOOD)

- If (OBS_CURRENT) > threshold**_jump**, then OBS_CURRENT is SUSPECT

Values of range**_jump** and threshold**_jump** pre-defined for each of the pollutant are the following :

**pollutant** **range_jump threshold_jump**

O_3_  60 ppbv 200 ppbv

NO_2_  30 ppbv 100 ppbv

PM_2.5_ 90 µg/m^3^ 200 µg/m^3^

PM_10_ 90 µg/m^3^ 200 µg/m^3^

SO_2_ 30 ppbv 100 ppbv

Suspicious values have flag which are changed as follow :

FLAG = FLAG|8 if SUSPECT => (bit3 = 2**3 = 8)

**Third QC - QC in the BGCKSFC (Background Check surface)**

1. **O-P Test**

The background check is applied to all Canadian and US observations which have been labeled as GOOD for all the pollutants considered

Algorithm description:

If (OBS_CURRENT is not GOOD) or (FORECAST < 0) or ( | O-P | > **range OmP**)

- then OBS_CURRENT is rejected

Values of range OmP are pre-defined for each of the following pollutants :

**pollutant** range OmP

O_3_ 50 ppbv

PM_2.5_ 100 µg/m^3^

PM_10_ 100 µg/m^3^

NO_2_ 30 ppbv

NO 30 ppbv

SO_2_ 30 ppbv

Note that rejected observations are not ingested in the OA. The FLAG value for observations are changed accordingly :

FLAG = FLAG|512|65536 🡺 512 means rejected station by QC (from objective analysis)

(bit9 = 2**9 = 512)

🡺 65536 gives the reason of reject: i.e. by comparison with the background field (bit16 = 2**16 = 65536)

1. **Tests for station location**

if (STATION outside model domain then the spatial interpolation is impossible)

- then STATION is rejected 🡺 STATUS|32 🡺 32 means station outside domain
- if (STATION not present in the error stats file)
- then STATION rejected 🡺 FLAG|2048 🡺 2048 means that the station is rejected by a selection process (bit11 = 2**11 = 2048) or was pre-identified on a black list.
