# Supplementary material for: Multi-pollutant surface objective analyses and mapping of air quality health index over North America
Source: Air Qual Atmos Health. 2016 Jan 7;9(7):743–59. doi: 10.1007/s11869-015-0385-9 (PMC5054062; doi:10.1007/s11869-015-0385-9)
Supplement: Supplementary file 2 — (PPTX 260 kb) [file 11869_2015_385_MOESM2_ESM.pptx]

## Slide 1
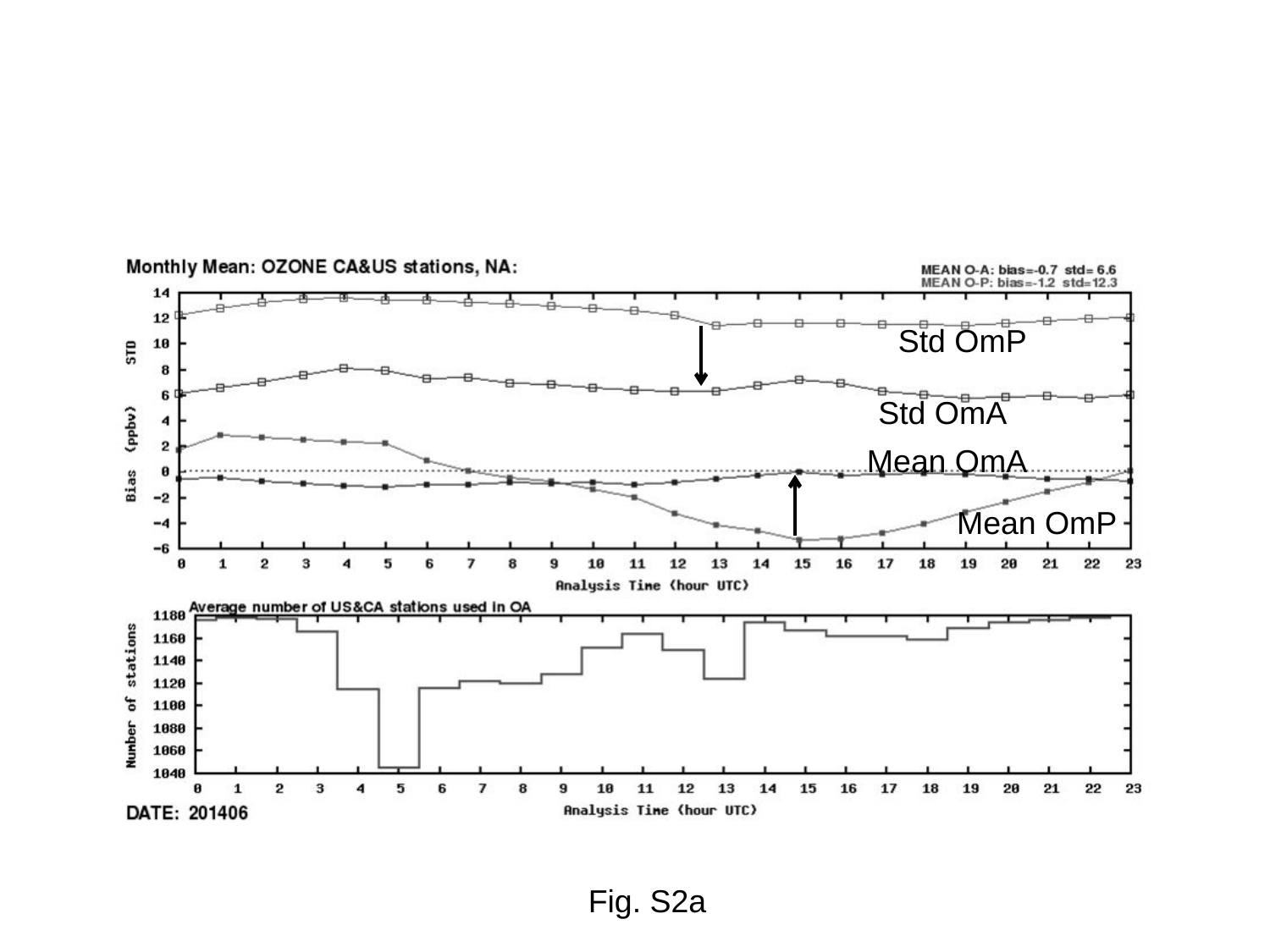

Std OmP
Std OmA
Mean OmA
Mean OmP
Fig. S2a

## Slide 2
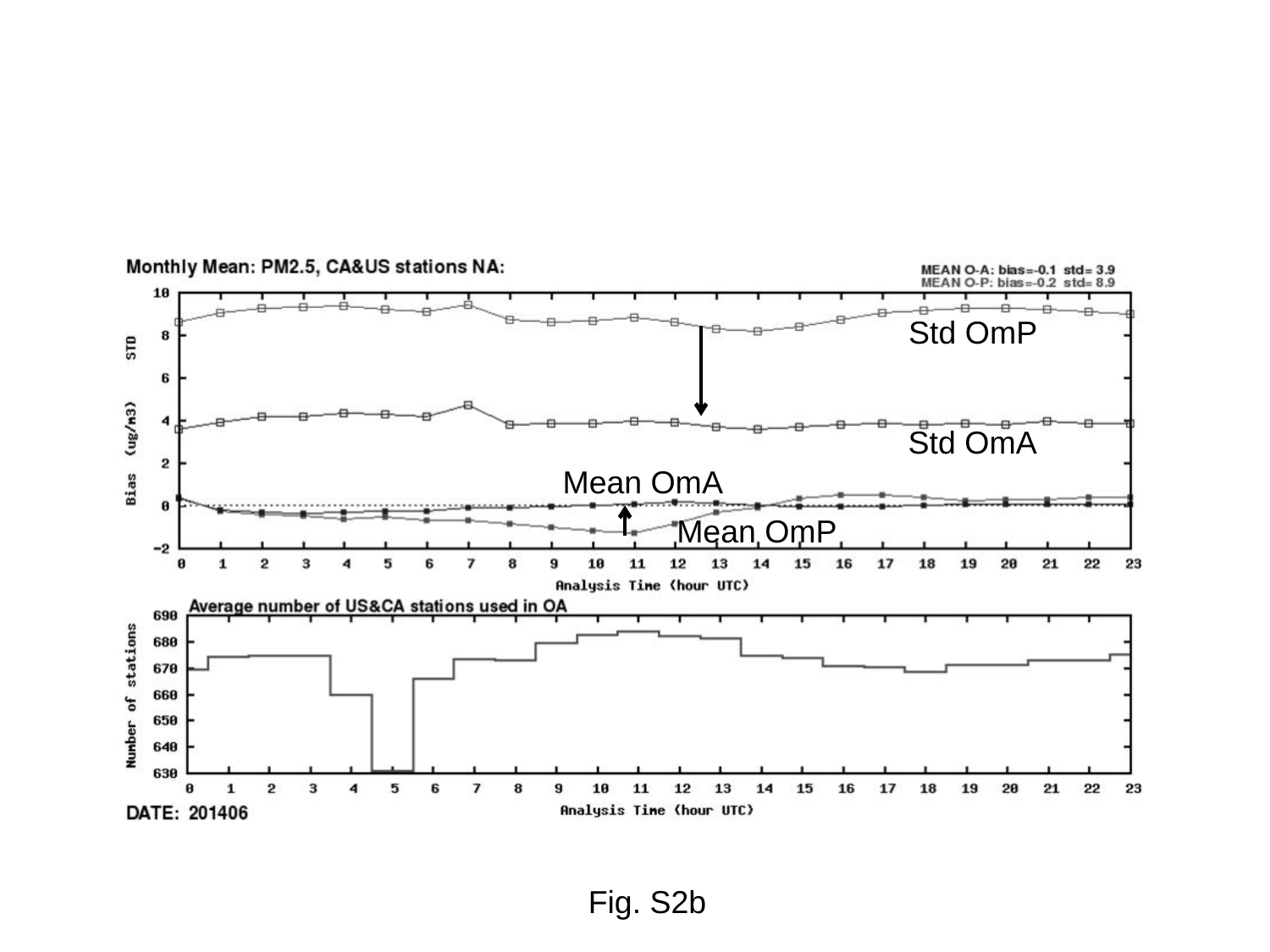

Std OmP
Std OmA
Mean OmA
Mean OmP
Fig. S2b

## Slide 3
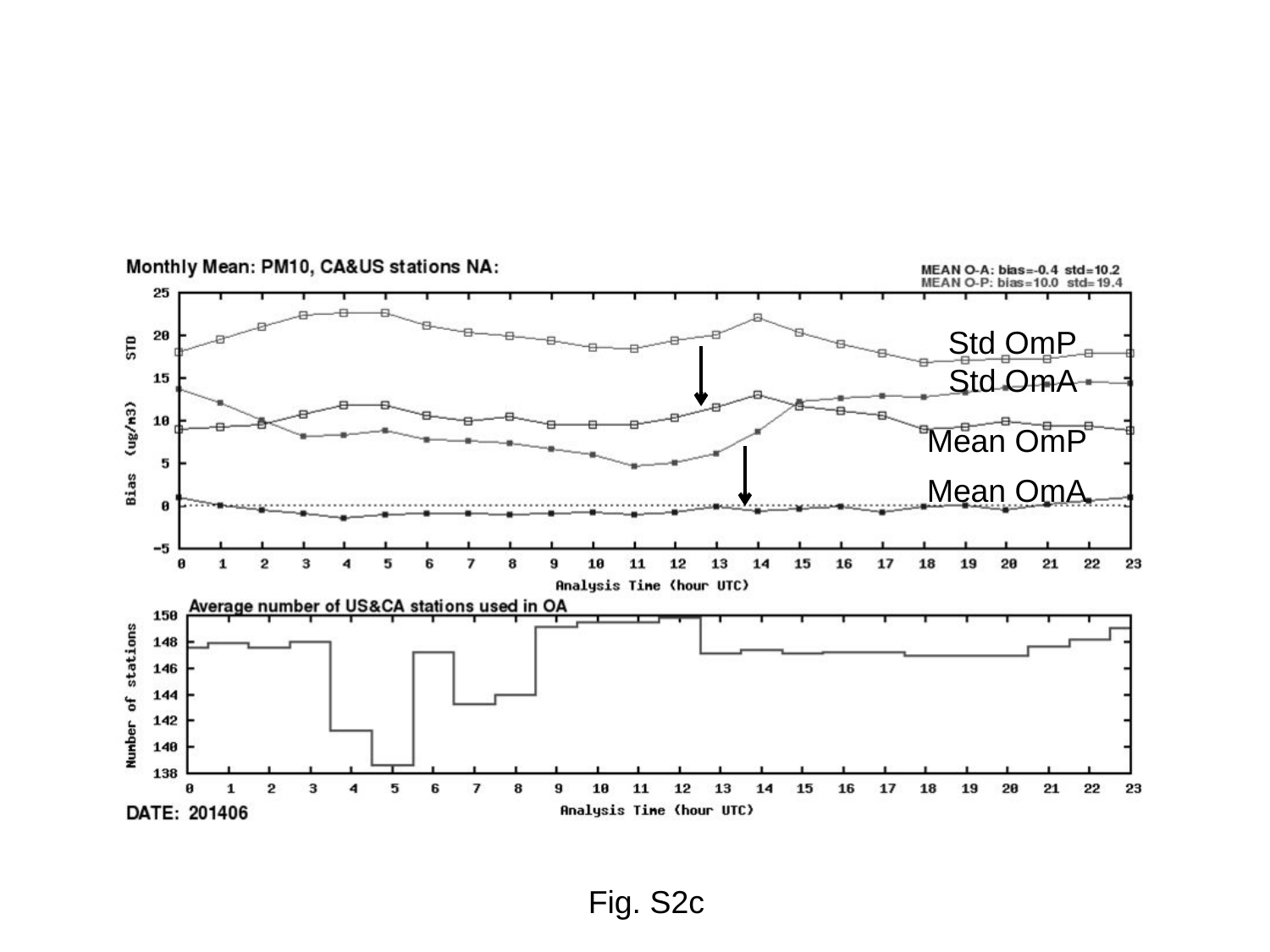

Std OmP
Std OmA
Mean OmP
Mean OmA
Fig. S2c

## Slide 4
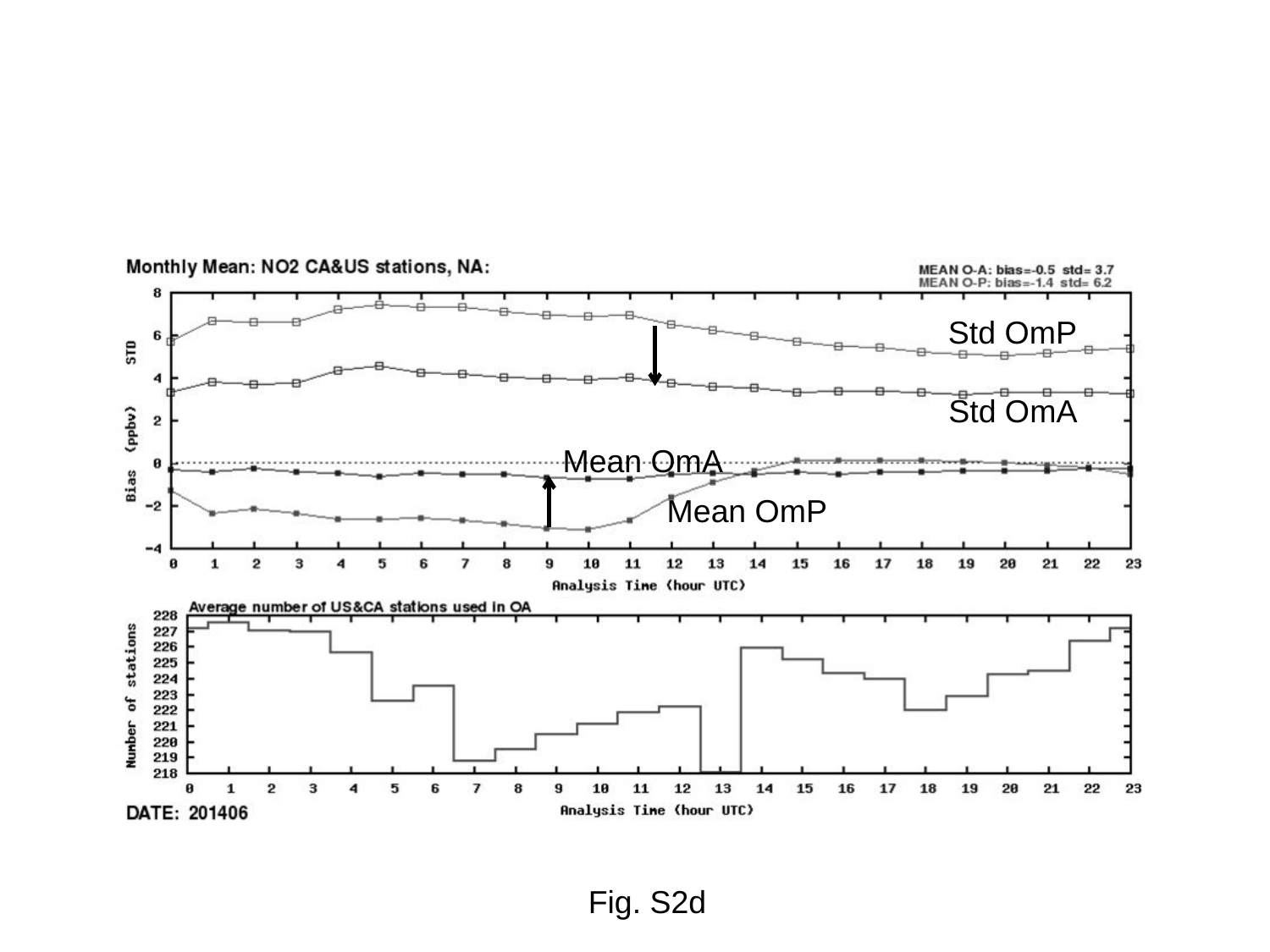

Std OmP
Std OmA
Mean OmA
Mean OmP
Fig. S2d
